# Association between quality of life and mental stress-induced myocardial ischaemia in high-risk patients after coronary revascularization

**DOI:** 10.1186/s12955-022-01976-0

**Published:** 2022-04-26

**Authors:** Nan Nan, Wei Dong, Bingyu Gao, Feihuan Cui, Zhi Chang, Jian Jiao, Huijuan Zuo, Hongzhi Mi, Xiantao Song, Shuzheng Lyu, Hongjia Zhang

**Affiliations:** 1 Department of Cardiology, Beijing Anzhen Hospital, Capital Medical University, No. 2, Anzhen Road, Chaoyang District, Beijing, 100029 China; 2 Department of Nuclear Medicine, Beijing Anzhen Hospital, Capital Medical University, Beijing, China; 3 Department of Psychology, Beijing Anzhen Hospital, Capital Medical University, Beijing, China; 4 Department of Community Health Research, Beijing Anzhen Hospital, Capital Medical University, Beijing Institute of Heart Lung and Blood Vessel Disease, Beijing, China; 5 Department of Cardiovascular Surgery, Beijing Anzhen Hospital, Capital Medical University; Beijing Lab for Cardiovascular Precision Medicine; Key Laboratory of Medical Engineering for Cardiovascular Disease, Capital Medical University, Beijing, China

**Keywords:** Mental stress-induced myocardial ischaemia, Quality of life, Seattle angina questionnaire, Coronary artery disease

## Abstract

**Objective:**

We sought to determine the association between mental stress-induced myocardial ischaemia (MSIMI) and quality of life (QoL) in patients with coronary artery disease (CAD) after coronary revascularization.

**Methods:**

This cohort study involved patients with high-risk MSIMI who received coronary revascularization between Dec 2018 and Dec 2019. Patients who screened positive for depression/anxiety were enrolled in this study. Mental stress was induced by the Stroop Colour and Word Test 1 month after coronary revascularization. All participants underwent single photon emission computed tomography (SPECT) scans at rest and under mental stress. MSIMI was defined as the presence of four abnormal SPECT phenomena. QoL was assessed using the Seattle Angina Questionnaire (SAQ) prior to treatment and 1 month after coronary revascularization.

**Results:**

Of the 1845 consecutive patients who received coronary revascularization, 590 (31.9%) had depression/anxiety, and 205 agreed to accept the mental stress test. During the average observation period of 33 days, 105 (51.2%) patients exhibited MSIMI. All SAQ subscales showed significant improvement, except for QoL, in the MSIMI group. The QoL score was lower (− 0.2 ± 32.7 vs. 13.1 ± 29.9, *P* = 0.005), and the proportion of deterioration in QoL was higher (50.5% vs. 31.9%, *P* = 0.010) in the MSIMI group than in the non-MSIMI group. Those with a deterioration in QoL had approximately twice the rate of MSIMI than those with an improvement in QoL (unadjusted OR: 2.019, 95% CI 1.122–3.634, *P* = 0.026; adjusted OR: 1.968, 95% CI 1.083–3.578, *P* = 0.017).

**Conclusion:**

Among patients with CAD who received coronary revascularization and had depression/anxiety, deterioration in QoL increased the likelihood of MSIMI. Hence, our results indicate that deterioration in QoL is a predictor of MSIMI.

*Trail Registration* ChiCTR2200055792, retrospectively registered, 2022.1.20, www.medresman.org.cn

## Introduction

Mental stress-induced myocardial ischaemia (MSIMI) is defined as an imbalance between myocardial oxygen supply and demand during mental or psychological stress. The data demonstrate that mental stress triggers transient myocardial ischaemia in 30–70% of patients with pre-existing coronary artery disease (CAD) [1–3]. To date, there are no guidelines on MSIMI, and numerous studies have found a higher prevalence and likelihood of MSIMI in patients with depression/anxiety. [4, 5]. The prevalence of MSIMI in patients with depression/anxiety and CAD was 22.08 times higher than for patients without depression/anxiety [4]. In addition, studies have also shown that a 1-point increase in the anxiety score is associated with a 1.22-fold increase in the likelihood of MSIMI, and a 5-point increase in the depression score is associated with a roughly twofold increase in the likelihood of MSIMI [4, 5].

The Seattle Angina Questionnaire (SAQ) is specific for CAD and has been widely accepted as a means of quantifying the outcome of CAD treatment on patients’ angina and QoL in trials [6]. The SAQ measures five subscales related to CAD, namely, physical limitations, angina frequency, angina stability, treatment satisfaction, and QoL. Among the MSIMI trials, only the angina-frequency subscale has been found to be related to MSIMI in women with stable CAD [7] and in post-myocardial infarction patients [8]. However, a comprehensive assessment of the five SAQ subscales, especially QoL and MSIMI, is limited.

Therefore, this study was designed to examine the association between SAQ and MSIMI in high-risk patients with depression/anxiety prior to revascularization and whose CAD stabilized after at least 1 month of revascularization. In addition, the association between MSIMI and the benefit or drawback of CAD revascularization assessed by SAQ was evaluated.

## Methods

### Study sample

In the study of CAD with a high risk of MSIMI patients, 1845 patients with coronary angiography-confirmed CAD who received coronary revascularization treatment, including percutaneous coronary intervention (PCI) or coronary artery bypass grafting (CABG), were screened between Dec 2018 and Dec 2019. The mental health status of all patients was assessed using the nine-item Patient Health Questionnaire-9 (PHQ-9) and the seven-item Generalized Anxiety Disorder-7 (GAD-7) severity measure. Those with screened depression/anxiety (PHQ-9 ≥ 5 and/or GAD-7 ≥ 5) were enrolled in this study. The study protocol was approved by the Medical Ethics Committee of Beijing Anzhen Hospital (NO. 2019001), and all participants provided informed consent prior to inclusion in this study.

### Mental stress test

Mental stress was induced by the Stroop Colour and Word Test (SCWT), which was employed to assess the ability to inhibit cognitive interference. The SCWT has been well accepted for assessing patients with CAD and has been shown to exhibit good reproducibility and execution [9, 10]. A standardized computer program was used to test the participants. Four words were displayed on the computer screen (i.e., “RED,” “BLUE,” “GREEN,” AND “YELLOW”), and corresponding buttons were labelled with one of four letters (i.e., “J,” “K,” “L,” and “I”). The computer randomly assigned the four colours to these four words, and only one word appeared at a time. The participant was instructed to match the word with the corresponding button without being distracted by the colour. For example, for the word “RED” presented in yellow, “J” should be selected rather than “I”. If the answer was wrong, the word “ERROR” appeared on the screen, and if the participant responded not quickly enough, the screen displayed the words “RESPOND FASTER”. During the test, an auditory stimulus was initiated by the examiners. The accuracy, response time, stimulus presentation velocity, and test duration were recorded by the computer. During the continuum of visual stimuli, a cognitive mechanism was applied to direct attention. The procedure included the following: (1) the participant was informed about the testing process and underwent a practice test that lasted approximately 1 min and then (2) the participant underwent a timed test. The SCWT lasted approximately 5 min. Non-invasive continuous blood pressure and heart rate measurements were performed every minute. The rate–pressure product (RPP) was calculated as systolic blood pressure (SBP) × heart rate (HR). The maximum values of SBP and HR during mental stress were selected.

### Single photon emission computed tomography (SPECT) myocardial perfusion imaging

Each participant underwent electrocardiographically gated technetium-99 m (^99m^Tc)-sestamibi SPECT imaging 1 month after PCI/CABG. All participants underwent two SPECT scans on two separate days: the first one at rest and the second one after mental stress. The patients received ^99m^Tc-sestamibi at a dose of 20–25 mCi during the rest and stress phases. The SCWT mental stress test lasted approximately 5 min, and ^99m^Tc-sestamibi was intravenously administered 1 min after the test started. Images were acquired using an e-Cam Duet gamma camera (Siemens AG, Erlangen, Germany) 60–90 min after the intravenous administration of ^99m^Tc-sestamibi.

The SPECT images were individually analysed by two experienced readers blinded to patient data. The extent and severity of myocardial ischaemia were assessed using a 17-segment model with Quantitative Gated SPECT software. Tridimensional reconstruction of the left ventricle was performed to assess ventricular function, in addition to the analyses of ejection fraction (EF), end-diastolic volume (EDV), end-systolic volume (ESV), contractility, and myocardial thickness [10]. Four abnormal SPECT phenomena were considered positive for MISIMI [11, 12], including reversible myocardial perfusion defects, transient ischaemic dilation (TID), reverse redistribution, and EF reduction of ≥ 5% [13]. The mental stress and rest images were analysed using total perfusion deficit (TPD) [14, 15].

### Assessment of coronary-related quality of life

The 19-item SAQ questionnaire, which measures five domains related to CAD, was used to assess angina frequency, physical limitations, angina stability, treatment satisfaction, and QoL. The scores ranged from 0 to 100, with higher scores indicating fewer symptoms and a better health status [16]. The participants were evaluated using the SAQ before revascularization and 1 month after revascularization. Significant improvements in each subscale were predefined for physical limitation (≥ 8 points), angina frequency (≥ 20 points), and QoL (≥ 16 points) [17].

### Statistical analysis

Continuous variables were reported as the mean ± standard deviation or median (Q1/Q3) when appropriate, and categorical variables were reported as numbers and percentages. Concomitant medication was summarized and compared between the groups at discharge and after 1 month using the χ^2^ test or Fisher’s exact test, whichever was appropriate. Each domain of the SAQ was evaluated using analysis of covariance before and after revascularization. Continuous variables were compared between groups using independent samples t test or Mann–Whitney-U Test. Within the same group, the differences in SAQ scores before and after revascularization were compared using a paired samples t test. For all other endpoints, a two-sided probability (p) value of 0.05 without correction for multiple testing was considered statistically significant. Logistic regression was used to identify the factors associated with MSIMI. The model was adjusted for coronary risk factors and imbalance factors before revascularization.

## Results

**Fig. 1:**
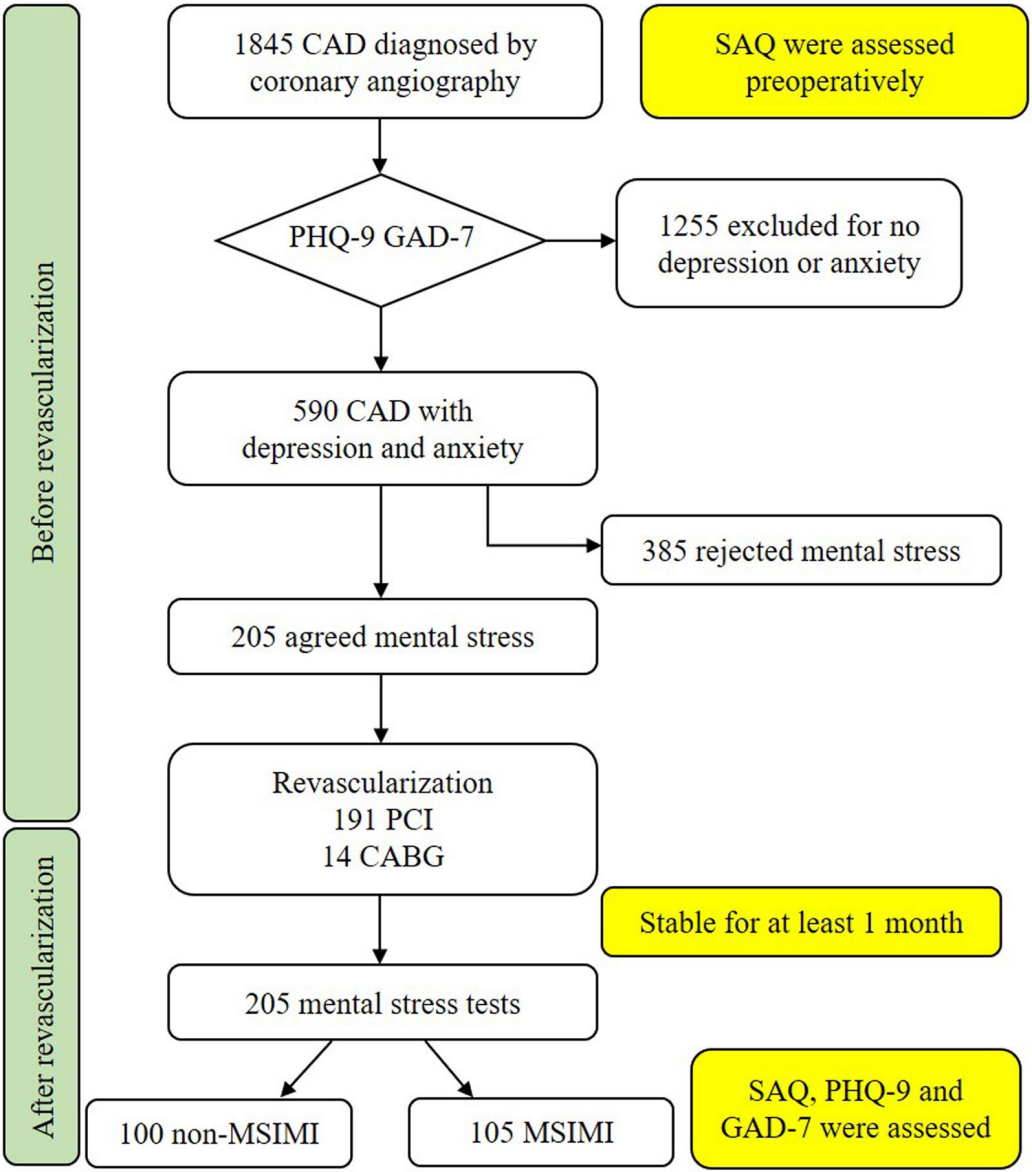
Flow chart of patient enrolment and the evaluation process

Of the 1,845 consecutive patients with CAD who received PCI/CABG, 590 (31.9%) exhibited depression/anxiety, and 205 agreed to undergo the mental stress test. Overall, 105 (51.2%) patients exhibited MSIMI. The enrolment and evaluation process are shown in the flow chart (see Fig. 1).

The mean age of the participants was 59.72 years (standard deviation [SD], 10.1), and 64 (31.2%) of the participants were women. The mental stress test was performed subsequent to an average observation period of 33 days (30/40) after coronary revascularization.

Among 105 patients exhibiting MSIMI, 59 (56.2%) had reversible myocardial perfusion defects, 30 (28.6%) had an EF reduction of ≥ 5%, 27 (25.7%) had TIDs, and 13 (12.4%) had reverse redistribution. Furthermore, 82 patients had only one abnormal phenomenon, 22 patients displayed a combination of two abnormal phenomena, and 1 patient exhibited three abnormal phenomena. The characteristics of the 205 patients before revascularization in the MSIMI and non-MSIMI groups are shown in Table 1. There were no significant differences between the two groups in the demographic risk factors or the angiographic severity of CAD. However, the MSIMI group demonstrated significantly higher PHQ-9 scores and longer disease duration while the non-MSIMI group had a higher drinking history percentage.

**Table 1:** Characteristics of the MSIMI and non-MSIMI groups before revascularization

	Non-MSIMI	MSIMI	Statistics	*P*
N	100	105(51.2)		
Age (years)	59.3 ± 10.0	60.2 ± 10.1	t = 0.626	0.532
Sex [female (%)]	25 (25.0)	39(37.1)	χ^2^ = 3.517	0.061
Hypertension (%)	63 (63.0)	72 (68.6)	χ^2^ = 0.707	0.400
Hyperlipidaemia (%)	59 (59.0)	65 (61.9)	χ^2^ = 0.181	0.671
Diabetes (%)	40 (40.0)	31 (29.5)	χ^2^ = 2.483	0.115
Depression history^a^ (%)	6 (6.0)	6 (5.7)	χ^2^ = 0.008	0.931
Current smoking (%)	38 (38.0)	32 (30.5)	χ^2^ = 1.289	0.256
Smoking history (%)	60 (60.0)	57 (54.3)	χ^2^ = 0.683	0.409
Drinking history (%)	55 (55.0)	40 (38.1)	χ^2^ = 5.886	**0.015**
Family history of CAD (%)	30 (30.0)	23 (21.9)	χ^2^ = 1.751	0.186
Disease duration^b^ (month)	6.0 (1.0/36.0)	18.0 (2.0/66.0)	U = 2.505	**0.012**
Number of events^c^	0.0 (0.0/1.0)	0.0 (0.0/1.0)	U = 1.819	0.069
SYNTAX score	17.0 (8.0/29.8)	18.0 (10.5/28.0)	U = 0.775	0.438
Residual SYNTAX	0.0 (0.0/3.0)	0.0 (0.0/7.5)	U = 1.804	0.071
Number of diseased blood vessels	2.0 (1.0/3.0)	2.0 (1.0/3.0)	U = 0.708	0.708
CAD type (%)			χ^2^ = 3.093	0.377
SAP	13 (13.0)	19 (18.1)		
UAP	62 (62.0)	69 (67.5)		
NSTEMI	9 (9.0)	5 (4.8)		
STEMI	16 (16.0)	12 (11.4)		
Revascularization type			χ^2^ = 3.824	0.281
PCI	94 (94.0)	97 (92.4)		
CABG	6 (6.0)	8 (7.7)		
PHQ-9 before revascularization	6.6 ± 3.1	7.9 ± 4.3	t = 2.368	**0.019**
GAD-7 before revascularization	5.1 ± 4.3	6.1 ± 5.0	t = 1.511	0.132
PHQ-9 after revascularization	3.7 ± 3.3	3.7 ± 3.3	t = 0.071	0.943
GAD-7 after revascularization	2.4 ± 3.0	2.3 ± 2.5	t = 0.394	0.694

MSIMI: mental stress-induced myocardial ischaemia; CAD: coronary artery disease; SAP: stable angina pectoris; UAP: unstable angina pectoris; NSTEMI: non-ST elevated myocardial infarction; STEMI: ST elevated myocardial infarction. PHQ-9: Patient Health Questionnaire-9; GAD-7: Generalized Anxiety Disorder-7; SYNTAX: Synergy between PCI with Taxus and Cardiac Surgery. *P* values < 0.05 are shown in bold

^a^Depression history means patients have been diagnosed with depression in their medical history

^b^Disease duration means the earliest onset time from symptom onset to the time of hospitalization (in months)

^c^Number of events refers to how many cardiovascular events (including non-fatal myocardial infarction, any coronary revascularization) the patient experienced throughout the course of the disease

Before mental stress, at rest, the left ventricle EDV and ESV were lower in the MSIMI group than in the non-MSIMI group (79.2 ± 30.1 vs. 92.7 ± 44.1 and 30.1 ± 21.7 vs. 40.4 ± 34.8, respectively, *P* < 0.05). Furthermore, EF was higher in the MSIMI group than in the non-MSIMI group (64.8 ± 12.8 vs. 60.0 ± 12.4, *P* < 0.01). On the day of the mental stress screening, there were no significant differences in EDV, ESV, or EF between the two groups, except for the higher proportion of stress TPD in the MSIMI group. Moreover, non-invasively measured SBP, DBP, HR, and RPP were similar in the two groups both at rest and during the mental stress test (see Table 2). None of the patients developed chest pain during the test.

**Table 2:** Differences in the indices between the non-MSIMI and MSIMI groups before and after mental stress

	Non-MSIMI	MSIMI	Statistics	*P*
Number	100	105(51.2)		
Rest status				
Rest TPD (%)	4.9 ± 11.0	4.12 ± 9.4	t = 0.551	0.582
Rest EDV (ml)	92.7 ± 44.1	79.2 ± 30.1	t = 2.577	**0.011**
Rest ESV (ml)	40.4 ± 34.8	30.1 ± 21.7	t = 2.517	**0.013**
Rest LVEF (%)	60.0 ± 12.4	64.8 ± 12.8	t = 2.707	**0.007**
Rest SBP (mmHg)	128.7 ± 16.7	131.4 ± 18.2	t = 1.083	0.280
Rest DBP (mmHg)	80.1 ± 11.0	78.2 ± 10.3	t = 1.262	0.208
Rest HR (bpm)	71.1 ± 10.8	69.8 ± 12.7	t = 0.791	0.430
Mental stress status				
Stress TPD (%)	4.8 ± 11.0	7.7 ± 9.8	t = 1.988	**0.048**
Stress EDV (ml)	91.3 ± 42.2	82.4 ± 29.2	t = 1.746	0.082
Stress ESV (ml)	38.2 ± 34.9	31.7 ± 21.6	t = 1.588	0.114
Stress LVEF (%)	62.4 ± 13.2	64.1 ± 12.0	t = 0.950	0.343
Stress max SBP (mmHg)	149.7 ± 18.7	150.4 ± 20.0	t = 0.273	0.785
Stress max DBP (mmHg)	90.2 ± 12.3	88.1 ± 11.0	t = 1.293	0.198
Stress max HR (bpm)	76.7 ± 12.6	74.1 ± 13.7	t = 1.362	0.175
RPP	11,518.4 ± 2709.1	11,150.0 ± 2528.3	t = 1.007	0.315

MSIMI: mental stress-induced myocardial ischaemia; TPD: total perfusion deficit; EDV: end-diastolic volume; ESV: end-systolic volume; LVEF: left ventricular ejection fraction; SBP: systolic blood pressure; DBP: diastolic blood pressure; HR: heart rate; RPP: rate–pressure product. *P* values < 0.05 are shown in bold

**Fig. 2:**
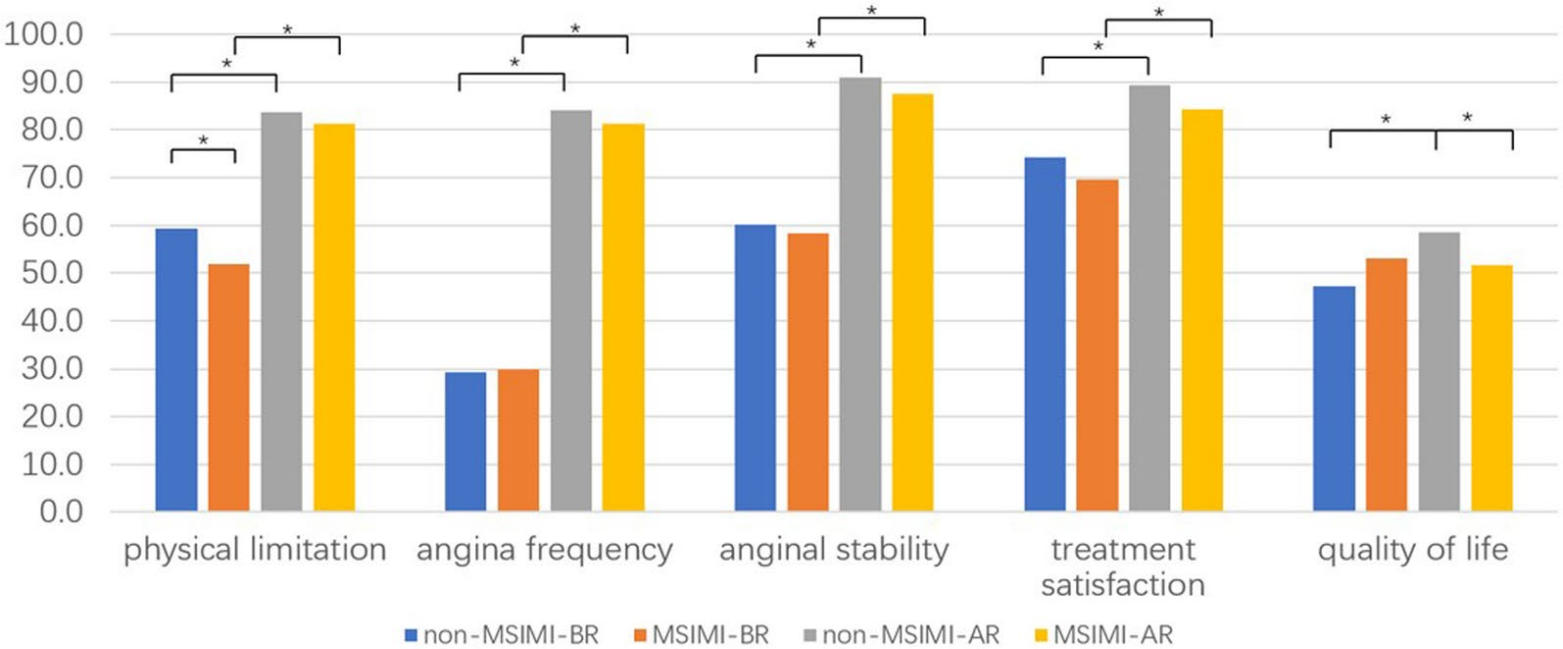
Change in the SAQ results of the non-MSIMI and MSIMI groups before and after coronary revascularization (*P < 0.05)

The five subscales measured using the SAQ questionnaire were similar between the non-MSIMI and MSIMI groups before revascularization. The only exception was physical limitations, which were significantly lower in the MSIMI group than in the non-MSIMI group (52.4 ± 20.2 vs. 58.7 ± 17.6, *P* = 0.025, see Table 3). At 1 month after coronary revascularization, all subscales were significantly improved in both groups (*P* < 0.001), except for the QoL subscale in the MSIMI group. Although the QoL improved significantly in the non-MSIMI group after coronary revascularization, the score was numerically but not statistically lower in the MSIMI group (see Fig. 2). Moreover, the QoL score was lower in the MSIMI group than in the non-MSIMI group after revascularization (51.5 ± 22.4 vs. 58.4 ± 22.8, *P* = 0.031).

**Table 3:** Five domains of the SAQ prior to treatment and 1 month after coronary revascularization in the non-MSIMI and MSIMI groups

	Non-MSIMI (n = 100)			MSIMI (n = 105)			*P*	
	BR	AR	*P*	BR	AR	*P*	BR-BR	AR–AR
Physical limitations	58.7 ± 17.6	83.8 ± 9.1	** < 0.001**	52.4 ± 20.2	81.4 ± 12.0	** < 0.001**	**0.025**	0.110
Angina frequency	29.0 ± 26.9	84.3 ± 24.5	** < 0.001**	30.2 ± 32.8	81.2 ± 24.5	** < 0.001**	0.788	0.372
Angina stability	60.1 ± 29.2	90.9 ± 15.1	** < 0.001**	58.2 ± 28.9	87.9 ± 17.4	** < 0.001**	0.663	0.139
Treatment satisfaction	73.6 ± 35.7	89.3 ± 32.7	** < 0.001**	70.0 ± 17.0	84.2 ± 13.2	** < 0.001**	0.383	0.145
Quality of life	46.7 ± 22.5	58.4 ± 22.8	** < 0.001**	53.6 ± 23.7	51.5 ± 22.4	0.939	0.087	**0.031**

BR: before revascularization; AR: after revascularization; SAQ: Seattle Angina Questionnaire; MSIMI: mental stress-induced myocardial ischaemia. *P* values < 0.05 are shown in bold

**Fig. 3:**
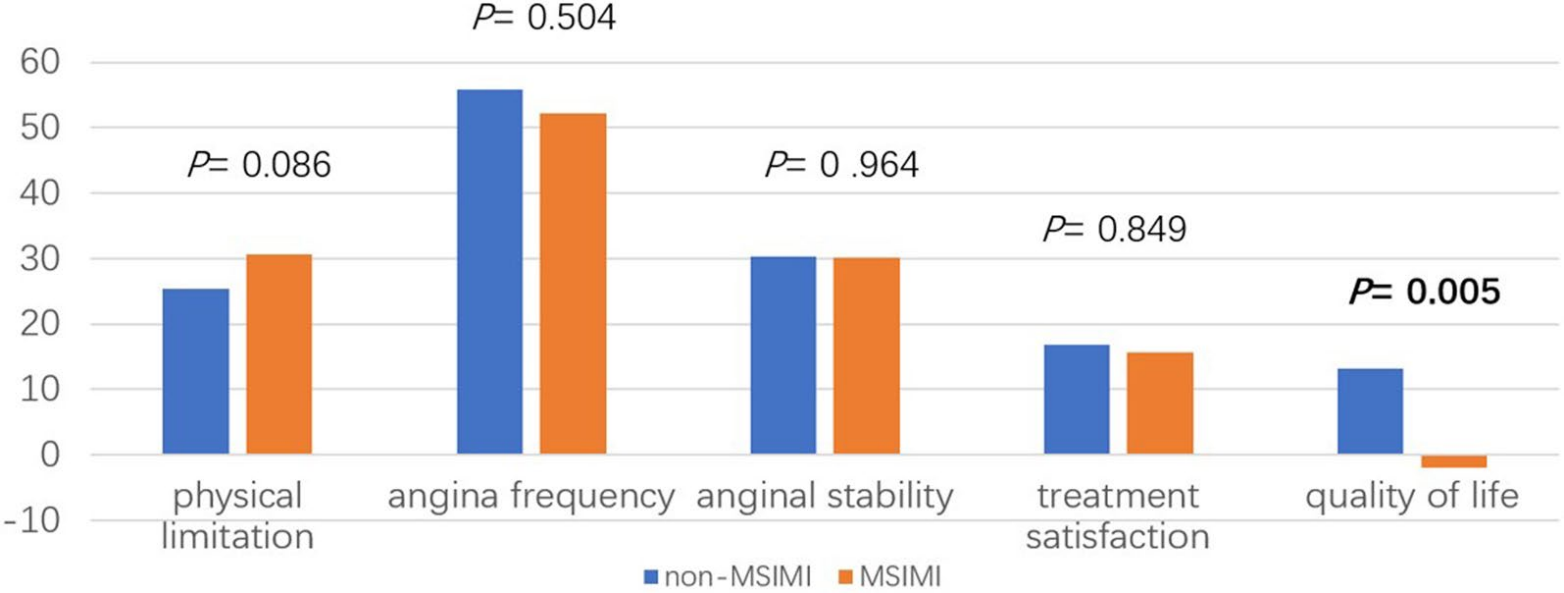
Comparisons of the SAQ improvements between the MSIMI and non-MSIMI groups after coronary revascularization

Table 4 shows the improvements in the five domains of the SAQ questionnaire. The absolute values of the improvements in physical limitations, angina frequency, angina stability, and treatment satisfaction were similar between the two groups. However, the improvement in QoL showed a significant difference between the two groups (13.1 ± 29.9 vs. − 0.2 ± 32.7, *P* = 0.005, see Fig. 3). More patients in the MSIMI group presented a significant improvement in the physical limitation subscale (87.9% vs. 76.3%, *P* = 0.041), and fewer patients in the MSIMI group showed a significant improvement in the QoL subscale (31.9% vs. 50.5%, *P* = 0.010). Overall, QoL showed a consistently deteriorating trend in the MSIMI group but not in the non-MSIMI group.

**Table 4:** Association between the improvements in SAQ and MSIMI

	Non-MSIMI	MSIMI	Statistics	*P*
Physical limitations				
Improvement value	25.4 ± 21.0	30.7 ± 20.7	t = 1.725	0.086
Has been improved	83 (89.2)	86 (94.5)	χ^2^ = 1.698	0.192
Improved ≥ 8	71 (76.3)	80 (87.9)	χ^2^ = 4.182	**0.041**
Angina frequency				
Improvement value	55.9 ± 34.5	52.2 ± 40.6	t = 0.670	0.504
Has been improved	82 (88.2)	71 (78.0)	χ^2^ = 3.382	0.066
Improved ≥ 20	82 (88.2)	71 (78.0)	χ^2^ = 3.382	0.066
Angina stability				
Improvement value	30.3 ± 32.1	30.1 ± 32.5	t = 0.045	0.964
Has been improved	70 (75.3)	69 (75.8)	χ^2^ = 0.008	0.930
Freedom from angina	57 (61.3)	51 (56.0)	χ^2^ = 0.522	0.470
Treatment satisfaction				
Improvement value	16.8 ± 49.9	15.7 ± 20.0	t = 0.190	0.849
Has been improved	69 (74.2)	65 (71.4)	χ^2^ = 0.178	0.673
Quality of life				
Improvement value	13.1 ± 29.9	− 0.2 ± 32.7	t = 2.863	**0.005**
Has been improved	57 (61.3)	40 (44.0)	χ^2^ = 5.545	**0.019**
Improved ≥ 16	47 (50.5)	29 (31.9)	χ^2^ = 6.613	**0.010**

SAQ: Seattle Angina Questionnaire; MSIMI: mental stress-induced myocardial ischaemia. *P* values < 0.05 are shown in bold

In the cohort that had CAD associated with depression/anxiety, the probability of MSIMI after 1 month of coronary revascularization was approximately two times higher in patients with a deterioration in QoL than in those with an improvement in QoL (unadjusted odds ratio [OR]: 2.019, 95% confidence interval [CI] 1.122–3.634; adjusted OR: 1.968, 95% CI 1.083–3.578). Similarly, an improved QoL score of < 16 was associated with a twofold increase in the probability of MSIMI when compared with a score of ≥ 16 (unadjusted OR: 2.184, 95% CI 1.199–3.979; adjusted OR: 2.105, 95% CI: 1.145–3.873) (see Table 5). Furthermore, a score of < 8 on the improved physical limitation subscale was associated with a 55.6% lower probability of MSIMI when compared with a score of ≥ 8 (unadjusted OR: 0.444, 95% CI 0.201–0.979); however, after adjusting for the factors before revascularization, the association with MSIMI did not exhibit statistical significance. In addition, although the decrease in angina frequency had no association with MSIMI, after adjustment, it was associated with a 2.336 times higher probability of MSIMI (adjusted OR: 2.336, 95% CI 1.029–5.301). For the other two subscales, namely, angina stability and treatment satisfaction, the association between deterioration or no significant improvement and MSIMI had no statistical significance.

**Table 5:** Association between the deterioration or no significant improvement in the SAQ subscales and MSIMI

	Unadjusted OR	*P*	Adjusted OR ^f^	*P*
Physical limitations				
Deterioration^a^	0.483 [0.158–1.472]	0.200	0.453 [0.145–1.420]	0.174
Improved < 8^b^	0.444 [0.201–0.979]	**0.044**	1.080 [0.992–1.175]	0.075
Angina frequency				
Deterioration^a^	2.100 [0.942–4.680]	0.070	2.336 [1.029–5.301]	**0.048**
Improved < 20^c^	2.100 [0.942–4.680]	0.070	1.078 [0.989–1.175]	0.086
Angina stability				
Deterioration ^a^	0.970 [0.495–1.901]	0.930	0.898 [0.450–1.791]	0.760
SAQ3 AR score < 100 ^d^	0.805 [0.447–1.449]	0.470	1.073 [0.986–1.167]	0.103
Treatment satisfaction				
Deterioration^a^	1.150 [0.600–2.203]	0.673	1.127 [0.581–2.188]	0.723
Quality of life				
Deterioration^a^	2.019 [1.122–3.634]	**0.019**	1.968 [1.083–3.578]	**0.026**
Improved < 16^e^	2.184 [1.199–3.979]	**0.011**	2.105 [1.145–3.873]	**0.017**

SAQ: Seattle Angina Questionnaire; MSIMI: Mental Stress-induced Myocardial Ischaemia; OR: Odds Ratio; AR: after revascularization. *P* values < 0.05 are shown in bold

^a^Deterioration vs. improvement in the SAQ results during the past 1 month

^b^Improvement in physical limitations of < 8 vs. significant improvement of ≥ 8

^c^Improvement in angina frequency of < 20 vs. significant improvement of ≥ 20

^d^SAQ3 FU score of < 100, denoting angina in the past 1 month, vs. freedom from angina

^e^Improvement in quality of life of < 16 vs. significant improvement of ≥ 16

^f^Adjusted for drinking history, PHQ-9 baseline, rest EDV, rest ESV, and rest LVEF

## Discussion

Among the group with a high risk of MSIMI, i.e., CAD with the comorbidity of depression/anxiety, coronary revascularization significantly improved physical limitations, angina frequency, angina stability, and treatment satisfaction but not QoL. The deterioration in QoL 1 month after coronary revascularization was associated with a two-fold increase in the risk of MSIMI. These findings were independent of traditional CAD risk factors and psychological factors.

Coronary revascularization, including PCI and CABG, has been proven to improve CAD-related SAQ scores in multiple populations. Our findings, except for QoL, are consistent with this observation. Among patients with stable CAD in the COURAGE study [18], SAQ was used to dynamically assess the QoL after PCI or optimal medical treatment (OMT). The greatest improvements were seen in the first 3 months, especially in the three subscales of physical limitations, angina frequency, and QoL. Among the patients with chronic coronary total occlusion lesions in the EUROCTO study [17], a greater improvement in the SAQ subscales was observed with PCI than with OMT for angina frequency and QoL. In a large sample of 3392 patients with CAD, coronary revascularization, regardless of the approach being PCI or CABG, was consistently associated with a significantly higher QoL, as assessed with SAQ at the one-year follow-up, when compared with other medical therapies [19]. In our study cohort of patients with CAD and depression/anxiety, the QoL improved in the non-MSIMI group but deteriorated in the MSIMI group 1 month after PCI or CABG. These findings indicate that MSIMI may weaken the beneficial effect of coronary revascularization on QoL.

In the MSIMI field, only two studies have thus far employed the SAQ to evaluate the relationship between CAD and MSIMI. In 950 patients with stable CAD, the angina frequency subscale of the SAQ was used to assess the probability of MSIMI. Overall, 338 individuals (37%) reported angina. It was found that only women who reported angina had MSIMI [7]. In our study, 47.3% of all patients reported angina, which is higher than that observed in the abovementioned study, but the decrease in angina frequency had only a weak association with MSIMI. This phenomenon could probably be attributed to the differences in the enrolment of participants. In our study, we considered depression/anxiety to be a high-risk factor for MSIMI that would interfere with the results [4, 20, 21]. Therefore, we enrolled patients with depression/anxiety. Previous studies have recorded that patients with depression who underwent revascularization demonstrated a lower improvement in angina frequency than those without depressive symptoms [22]. Consistent with our findings, another study involving 98 post-myocardial infarction patients found that the angina frequency score was positively associated with MSIMI after adjusting for depressive and anxiety symptoms [8]. However, neither study comprehensively evaluated the five subscales of the SAQ. To date, there have been no studies on the relationship between SAQ QoL and MSIMI.

The SAQ scores have been reported to be independently associated with 1-year cardiovascular events among patients with CAD and predict 1-year mortality and cardiac rehospitalizations [23, 24]. The deterioration in QoL might be related to many factors. Depression may lead to a decline in QoL [25]. In addition, depressive symptoms may aggravate the perception of chest pain and result in a worse QoL [22, 26]. Although cognitive behavioural therapy [22] or slow breathing therapy [27] appear to have at least modest benefits in improving QoL, studies examining antidepressant therapies have been inconclusive.

The main strength of our study is the selection of patients with a high risk for MSIMI. Previous studies have shown that depression/anxiety may be a risk factor for MSIMI [4, 20, 21]. Thus, we selected patients with CAD and reported depression/anxiety as the inclusion criterion to eliminate the influence of emotional factors on the results to the best possible extent. Furthermore, in previous MSIMI studies [7, 8, 28–30], CAD has mostly been determined based on medical history or self-reporting. However, all participants in our study were confirmed to have CAD via coronary angiography, which is more accurate than the methods used in previous studies. Moreover, considering that endothelial function and coronary microvascular disorders are possible mechanisms of MSIMI [31], except for myocardial perfusion defects, we selected TID, reverse redistribution, and EF reduction of ≥ 5%, which reflected microvascular disorders [11]. Additionally, we specifically evaluated the relationship between MSIMI and ischaemia-related vessels by analysing coronary angiography and SPECT images.

One of the key limitations of our study was the short follow-up period. The average follow-up time was only 33 days. In future research, we plan to follow up and obtain SAQ results at 3 months, 6 months, and 12 months to dynamically evaluate the association with MSIMI. In addition, we did not include the effects of MSIMI and major adverse cardiovascular events. In the future, we intend to follow-up on the events using repeat coronary angiography and psychological stress tests at 12 months.

## Conclusion

To the best of our knowledge, this is the first study demonstrating that deterioration in CAD-related QoL is associated with an increased likelihood of MSIMI among patients with CAD and depression/anxiety. Our results highlight the relationship between QoL and the likelihood of MSIMI.

## Data Availability

The datasets analyzed during the current study are not publicly available because the data are guaranteed to be confidential.

## Abbreviations

MSIMI: Mental Stress-induced Myocardial Ischaemia
QoL: Quality of Life
CAD: Coronary Artery Disease
SAQ: Seattle Angina Questionnaire
SPECT: Single Photon Emission Computed Tomography
OR: Odds Ratio
CI: Confidence Interval
PCI: Percutaneous Coronary Intervention
CABG: Coronary Artery Bypass Grafting
PHQ-9: Patient Health Questionnaire-9
GAD-7: Generalized Anxiety Disorder-7
SAP: Stable angina pectoris
UAP: Unstable angina pectoris
NSTEMI: Non-ST elevated myocardial infarction
STEMI: ST elevated myocardial infarction
SCWT: Stroop Color and Word Test
RPP: Rate-Pressure Product
SBP: Systolic Blood Pressure
DBP: Diastolic Blood Pressure
HR: Heart Rate
EF: Ejection Fraction
LVEF: Left Ventricular Ejection Fraction
EDV: End-Diastolic Volume
ESV: End-Systolic Volume
TID: Transient Ischaemic Dilation
TPD: Total Perfusion Deficit
SYNTAX: Synergy between PCI with Taxus and Cardiac Surgery
OMT: Optimal Medical Treatment
